# [U^III^{N(SiMe_2_*t*Bu)_2_}_3_]: A Structurally Authenticated Trigonal Planar Actinide Complex

**DOI:** 10.1002/chem.201404864

**Published:** 2014-09-21

**Authors:** Conrad AP Goodwin, Floriana Tuna, Eric JL McInnes, Stephen T Liddle, Jonathan McMaster, Inigo J Vitorica-Yrezabal, David P Mills

**Affiliations:** [a] School of Chemistry, The University of ManchesterOxford Road, Manchester, M13 9PL (UK); [b] School of Chemistry, The University of NottinghamUniversity Park, Nottingham, NG7 2RD (UK)

**Keywords:** actinides, ligand design, ligand effects, single-molecule magnets, uranium

## Abstract

We report the synthesis and characterization of the uranium(III) triamide complex [U^III^(N**)_3_] [**1**, N**=N(SiMe_2_*t*Bu)_2_^−^]. Surprisingly, complex **1** exhibits a trigonal planar geometry in the solid state, which is unprecedented for three-coordinate actinide complexes that have exclusively adopted trigonal pyramidal geometries to date. The characterization data for [U^III^(N**)_3_] were compared with the prototypical trigonal pyramidal uranium(III) triamide complex [U^III^(N“)_3_] (N”=N(SiMe_3_)_2_^−^), and taken together with theoretical calculations it was concluded that pyramidalization results in net stabilization for [U^III^(N“)_3_], but this can be overcome with very sterically demanding ligands, such as N**. The planarity of **1** leads to favorable magnetic dynamics, which may be considered in the future design of U^III^ single-molecule magnets.

Investigations into low-coordinate metal complexes (defined herein as coordination number, CN<4) are legion, because they can exhibit interesting properties,^1^ including small-molecule activation chemistry^2^ and single-molecule magnet (SMM) behavior.^3^ Low CN complexes usually contain sterically demanding ligands to prevent oligomerization,^1^ in which bulky monodentate amides are frequently utilized.^4^ The bulky silylamide {N(SiMe_3_)_2_}^−^ (N“) has provided landmark low CN complexes; for example, three-coordinate [M^III^(N”)_3_] complexes of Group 13 (M=Al, Ga, In, Tl)^5^ and first row d-block (M=Ti–Co)^6^ metals are trigonal planar (*D*_3*h*_) in the solid state, but Group 3,6a, 7 lanthanide (Ln),^7^ and actinide (An)^8^ [M^III^(N“)_3_] complexes exhibit trigonal pyramidal (*C*_3*v*_) solid-state geometries, although they have zero dipole moment in solution, inferring that they may become planar in this phase.^9^ Pyramidal geometries persist for [Ln^III^(N”)_3_] (Ln=Ce, Pr) in the gas phase,^10^ but [Sc^III^(N“)_3_] vapors are *D*_3*h*_, with crystalline/gas-phase discrepancies for this complex attributed to crystal-packing effects.^11^ It is noteworthy that complexes, such as [Ln^II^(N”)(μ-N“)_2_Na] (Ln=Eu, Yb) and [Sm^II^(N”)(μ-N“)_2_M] (M=Na, K), have trigonal planar Ln coordination spheres,^[12]^ but this geometry has not been previously observed in An complexes.

f-Block metal centers favor high CNs, because Ln and An cations have relatively large ionic radii and bonding regimes that are dominated by electrostatic contributions.^13^ Low CN U^III^ chemistry is burgeoning, driven by interesting small molecule activation reactions^[14]^ and intrinsic SMM behavior.^15^ Structurally characterized three-coordinate An complexes to date adopt exclusively trigonal pyramidal geometries rather than trigonal planar or T shaped (*C*_2*v*_),^16^ although matrix isolation experiments^[17]^ and calculations^18^ have shown that monomeric UO_3_ is T shaped. Both covalent^[19]^ and electrostatic^10^ arguments account for the trigonal pyramidal geometry of [U^III^(N“)_3_],^8^, ^20^ hence, the most influential factor of these two for causing pyramidalization has never been established. Herein, we report the structurally characterized An complex, [U^III^(N**)_3_] (**1**, N**=N(SiMe_2_*t*Bu)_2_^−^), which adopts an unprecedented trigonal planar geometry for an actinide triamide complex. Complex **1** is closely related to [U^III^(N”)_3_], allowing the contributions to pyramidalization to be assessed, together with the impact of geometry on magnetic (including dynamic) and electronic properties of U^III^ complexes, for the future rational design of useful An materials.

Complex **1** was prepared by a modification of the revised synthesis of [U^III^(N“)_3_].8c Compound [U^III^(I)_3_(THF)_4_]8c was reacted with 1.5 equivalents of [K{N(SiMe_2_*t*Bu)_2_}]_2_ in THF, followed by work-up and recrystallization from hexane to give **1** as dark purple needles in 62 % yield (Scheme 1).^21^ Absorbances in the FTIR spectrum of **1** at 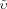, 825, and 761 cm^−1^ are attributed to the UNSi_2_ stretching modes of the silylamide ligand. The asymmetric stretch (950 cm^−1^) is 40 cm^−1^ lower than that observed for [U^III^(N”)_3_] (990 cm^−1^),8a which is of a similar magnitude to the differences between previously reported planar and pyramidal [M(N“)_3_] MNSi_2_ asymmetric stretches (ca. 50 cm^−1^).5b, 6a

**Scheme 1:**
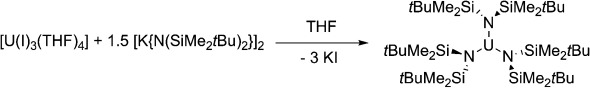
Synthesis of 1.

The ^1^H NMR spectrum of **1** exhibits two resonances at *δ*=3.8 (*ν*½=206 Hz) and −47.0 ppm (*ν*½=4597 Hz) in a 54:36 ratio that are assigned to the *t*BuSi and Me_2_Si protons, respectively. The Me_2_Si resonance of **1** is much broader than the analogous resonance for [U^III^(N“)_3_] (*δ* −11.4, *ν*½=15 Hz),^8^ but variable-temperature (VT) studies gave a sharper resonance at 353 K (*δ*=−32.9 ppm, *ν*½=266 Hz).^21^ A wide-scan ^13^C NMR spectrum of **1** exhibited two resonances for the Me_2_Si (*δ*=−2.1 and 1.5 ppm) and *t*BuSi quaternary carbons (*δ*=18.2 and 32.0 ppm), but only one for the *t*BuSi primary carbons (*δ*=26.4 ppm). In contrast, in the ^13^C NMR spectrum of [U^III^{N(SiPhMe_2_)_2_}_3_], the Me_2_Si group resonates at *δ*=−57.1 ppm.^22^ A resonance was observed in the ^29^Si NMR spectrum of **1** at *δ* −296.0 ppm (*ν*½=73 Hz), which has not been reported for similar systems,^8^, ^22^ but is typical for a U^III^ complex.^23^

The electronic absorption spectrum of **1**^21^ exhibited 5f^3^→5f^2^6d^1^ transitions at 20 000 (*ε*=776 m^−1^ cm^−1^) and 22 500 cm^−1^ (*ε*=770 m^−1^ cm^−1^) that are typical of U^III[24]^ and comparable to a broad absorption observed for [U^III^{N(SiPhMe_2_)_2_}_3_] at 21 500 cm^−1^ (*ε*=430 m^−1^ cm^−1^).^22^ In the 7 000–13 000 cm^−1^ region, weak Laporte forbidden 5f→5f transitions were observed (*ε*=15–64 m^−1^ cm^−1^).^25^ Similar weak absorptions were observed for most U^III^ complexes, such as [U(I)_3_(THF)_4_]8c, 26 and [U^III^{N(SiPhMe_2_)_2_}_3_],^22^ and strong absorptions in this region are very rare.^[27]^

The crystal structure of **1** was determined and is depicted in Figure 1, with selected metrical parameters.^28^ Complex **1** crystallizes in the *C*2/*c* space group, with a twofold axis bisecting the U(1)–N(1) bond. This contrasts to [Fe(N“)_3_],^9^ [Eu^III^(N”)_3_],^29^ [U^III^(N“)_3_],8d and [Pu^III^(N”)_3_],8e which all crystallize exclusively in the *P*3_1_*c* space group, and [U^III^{N(SiPhMe_2_)_2_}_3_], which crystallizes in *R*3.^22^ The U atom of **1** is almost ideally trigonal planar, with U–N bonds that are statistically identical within experimental uncertainty [U-N range 2.403(3)–2.415(6) Å]. These distances are longer than those observed in [U^III^(N“)_3_] [2.320(4) Å]8d and [U^III^{N(SiPhMe_2_)_2_}_3_] [2.34(2) Å],^22^ which can be attributed to the greater interligand repulsion in **1** arising from the sterically demanding *t*Bu groups. The U centroid/N(1)-N(2)-N(2A) mean plane distance in **1** is 0.008(2) Å, and the N-U-N bond angles (range 119.1(2)–120.47(9)°) sum to 360°; in contrast, [U^III^(N”)_3_] and [U^III^{N(SiPhMe_2_)_2_}_3_] exhibit U centroids 0.456(1) and 0.874 Å from the N_3_ planes, and the N-U-N angles average 116.24(7) (Σ angles 348.72(7)°) and 106.88° (Σ angles 320.64°), respectively.8d, 22 The UNSi_2_ fragments of **1** are essentially planar and all bisect the UN_3_ plane (range 53.23–61.35°) to form a molecular propeller.

**Figure 1:**
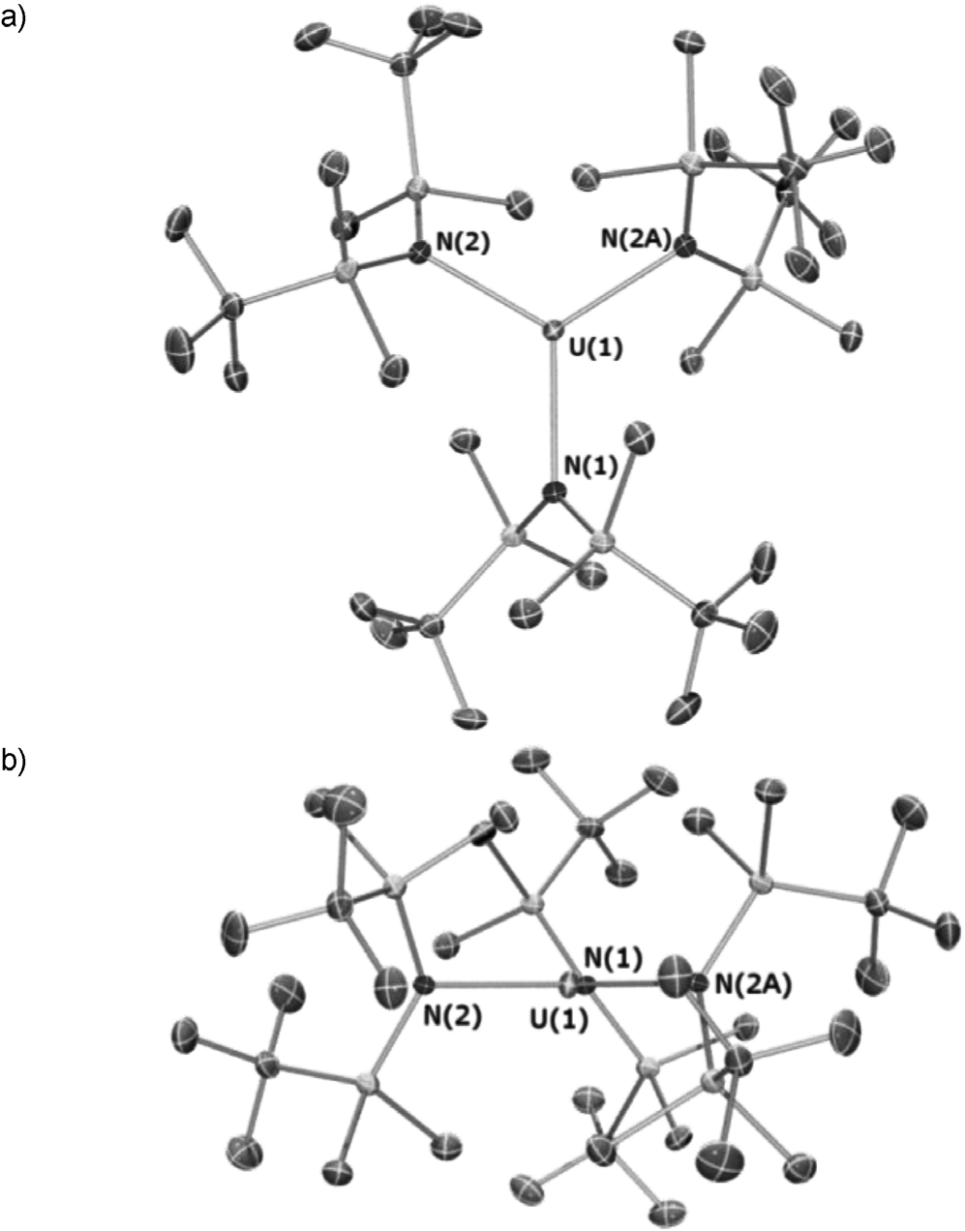
Molecular structures of 1 a) top view and b) along twofold axis, with selected atom labelling. Displacement ellipsoids are set at the 40 % probability level, and hydrogen atoms are removed for clarity. Selected bond lengths [Å] and angles [°]: U(1)–N(1) 2.403(3), U(1)–N(2) 2.415(6); N(1)-U(1)-N(1′) 119.05(19), N(1)-U(1)-N(2) 120.47(9).

The pyramidal geometries of [U^III^(N“)_3_] and [U^III^{N(SiPhMe_2_)_2_}_3_] are predicted by the polarized-ion model, whereby net stabilization was achieved by dipole formation.8d, 22 [U^III^(N”)_3_] exhibits unequal U-N-Si angles (108.50(7) and 125.25(7)°), because one Si–C bond for each N“ ligand is relatively close to the U center [U**⋅⋅⋅**C_γ_ 3.05 Å; U**⋅⋅⋅**Si 3.29 Å].8d These can be attributed to stabilizing agostic M**⋅⋅⋅**Si–C_γ_ interactions, as have been discussed for [U^III^{CH(SiMe_3_)_2_}_3_]^30^ and [Sm^III^(N”)_3_].^[31]^ The shortest U**⋅⋅⋅**C_γ_ and U**⋅⋅⋅**Si distances in **1** are 3.119–3.301 Å and 3.433–3.510 Å, respectively, and they are not correctly orientated to interact with the U center. Although there is no evidence for agostic U**⋅⋅⋅**Si–C_γ_ interactions in **1**, stabilizing U**⋅⋅⋅**C–H contacts cannot be discounted.

Unrestricted DFT calculations were carried out on full models of **1** and [U^III^(N“)_3_].^21^ The geometry-optimized structures reproduce the experimental structures with good agreement, despite the slight deviation from planarity for the model of **1** (discrepancies attributed to this being a gas-phase calculation, which does not account for crystal-packing forces), providing qualitative models (bond lengths within 0.05 Å, angles within 1°, U centroid/N_3_ mean plane distance: **1** 0.132 Å, [U^III^(N”)_3_] 0.393 Å). In both models, the HOMO, HOMO−1 and HOMO−2 represent the three unpaired U^III^ 5f electrons (**1**: 93.93, 94.71, 90.09; [U^III^(N“)_3_] 86.81, 86.32, 84.17 % U 5f, respectively). Both models exhibit essentially insignificant degrees of U 6d/5f orbital contributions to the U–N bonds, with the HOMO−3, HOMO−4. and HOMO−5, representing the π components (**1**: 5.27/0, 1.57/0, 0/1.31; [U^III^(N”)_3_] 4.29/0, 0/2.06, 1.63/1.39 % U 5f/6d, respectively) and the HOMO−6, HOMO−7, and HOMO−8 the σ components (**1**: 0/2.29, 0/2.12, 1.20/0; [U^III^(N“)_3_] 0/5.04, 0/5.26, 2.14/0 % U 5f/6d, respectively). This concurs with gas-phase photoelectron spectroscopy (PES) studies of [U(N”)_3_], which have shown that π bonding between the ligand and U center is insignificant in this complex.^32^ The calculated uranium spin densities (MDC-m α spin, **1**=−3.26; [U^III^(N′′)_3_]=−3.26) are identical, which also supports similar bonding patterns for **1** and [U^III^(N“)_3_].

Ab initio calculations on [An^III^(CH_3_)_3_] (An=U, Np, Pu)^33^ and [An^III^(NH_2_)_3_] (An=U, Np)^34^ have shown that the involvement of An 6d orbitals in the U–X (X=C, N) σ components may be associated with pyramidalization in the absence of steric contributions. Thus, given the similar bonding within **1** and [U^III^(N“)_3_] together with the small U 6d/5f contributions to the U–N σ and π components, we suggest that the experimentally determined trigonal planar geometry of **1** results from steric interactions involving the large N** ligands. These interactions could predominate over crystal packing forces, which are often only approximately 10 kJ mol^−1^.^35^ We conclude that there are minor differences in bonding between **1** and [U^III^(N”)_3_], therefore, the planar geometry of **1** derives principally from steric effects involving the ligands.

The solution magnetic moment of **1** was calculated to be 2.59 μ_B_ in [D_6_]benzene at 298 K by using the Evans method.^[36]^ Magnetometry measurements on a powdered sample of **1** suspended in eicosane gave a magnetic susceptibility temperature product, *χT*, of 1.07 cm^3^ Kmol^−1^ (2.92 μ_B_) at 298 K,^21^ which corresponds well with the solution measurement considering weighing errors and the difference in phase. These values are lower than for a free-ion 5f^3 4^I_9/2_ ground state (3.69 μ_B_), because not all crystal field levels are thermally occupied,^37^ but are typical for U^III^ complexes described in the literature (range 2.13–4.63 μ_B_).^8^, ^15^, ^22^, ^25^, ^26^, ^30^, ^[38]^ The *χT* value of **1** decreases to 0.41 cm^3^ Kmol^−1^ at 2 K; ac measurements give a low-temperature plateau in the in-phase *χ′T* at 0.48 cm^3^ Kmol^−1[21]^ consistent with thermal depopulation into a Kramers doublet ground state.^3^, ^13^ Low-temperature EPR spectra of **1** are consistent with U^III^,^[27]^ and simulation gives *g*_eff_=3.55, 2.97, and 0.553 for the ground Kramers doublet (the latter is observed at high field at X-band, but is beyond the magnetic field range at Q band; Figure 2 a).

**Figure 2:**
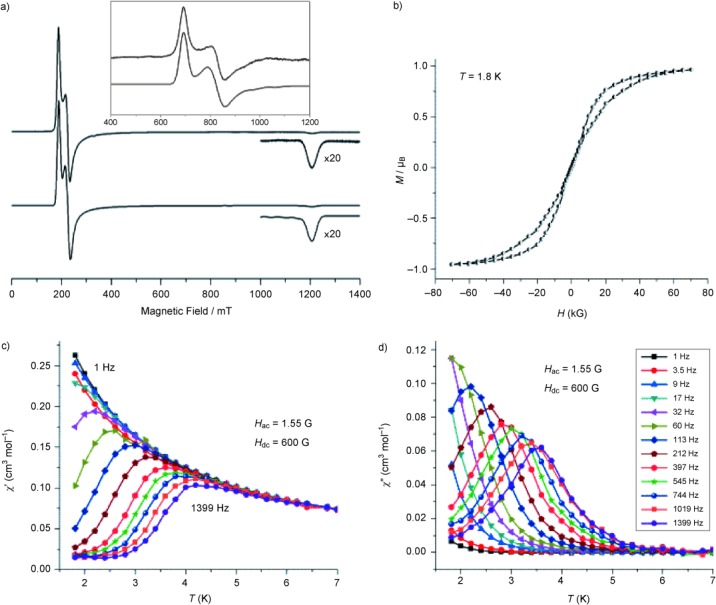
a) X- (9.5 GHz) and Q-band (34 GHz; inset) EPR spectra of 1 at 5 K. Lower spectra are simulations as *S*_eff_=1/2. Magnetic-susceptibility data for 1: b) magnetic hysteresis at 1.8 K, sweep rate 13 G s^−1^; c) in-phase (*χ′*); and d) out-of-phase (*χ*“) components of the ac susceptibility measured in an applied dc field of 600 G and an oscillating field of 1.55 G.

Compound [U^III^(N“)_3_] is an SMM,^15^ hence, we have performed low-temperature ac measurements on **1** to probe differences in the dynamic magnetic behavior as a result of the higher symmetry. Compound **1** is also an SMM, with clear frequency-dependent behavior (Figure 2 c and d).^21^ Under the optimal dc field of 600 G, the magnetization relaxes much slower than in [U^III^(N”)_3_], and maxima in the out-of-phase susceptibility *χ*′′(*T*) are seen to significantly higher temperatures for **1** than for [U^III^(N“)_3_] at equivalent frequencies (e.g., 3.5 vs. 2.1 K, respectively, for 1.4 kHz). An Arrhenius treatment^21^ of the higher-temperature ac data gives an energy barrier of *U*_eff_=21.4±0.2 K for **1**. Although this is lower than that reported for [U^III^(N”)_3_] (31 K), the latter value was derived from an extremely limited temperature range^15^ and should be treated with some caution. The relaxation time (*τ*) at 2 K is 2.6 ms for **1**; from the previously reported data^15^ we find 0.3 ms for [U^III^(N“)_3_] at 2 K, an order of magnitude quicker. The pre-factor *τ*_0_ for **1** is greater by four orders of magnitude (3.1×10^−7^ cf. 10^−11^ s for [U^III^(N”)_3_]).^15^ Moreover, the frequency dependence of *χ*′ and *χ*“ at 1.8 K for **1**^21^ reveal a single relaxation process with a narrow distribution in relaxation times (*α*=0.001–0.03 from Cole–Cole analysis), an order of magnitude lower than in [U^III^(N”)_3_] (*α*=0.09–0.34).^15^ In fact, the difference in dynamics is sufficient that magnetization hysteresis is observed for **1** at 1.8 K on a conventional superconducting quantum interference device (SQUID) magnetometer (Figure 2 b), while it is not for [U^III^(N“)_3_].

In the trigonal planar geometry of **1**, with no axial ligands, we expect a low *J_z_* state of U^III^ to be stabilized by the crystal field. This is supported by the EPR analysis: if we assume a ^4^I_9/2_ ground term,^39^ with *g*_J_=8/11, the *J_z_*=±1/2 doublet is calculated to have *g*_*x*,*y*_=3.65, *g_z_*=0.73 (all other doublets have *g*_*x*,*y*_=0), in good agreement with experiment. |*J_z_*|=1/2 is also the ground doublet of the (pyramidal) 4f^3^ complex [Nd^III^(N“)_3_] from optical studies.^40^ Hence, **1** and [U^III^(N”)_3_] are SMMs despite their easy-plane anisotropy: this highlights the complexity of interpreting f-block relaxation data,^41^ particularly when relatively low (tens of K) energy barriers are involved. At this stage, we can speculate that the “cleaner” and slower relaxation of **1** compared with [U^III^(N“)_3_] on flattening the geometry is because of quenched mixing. In *D*_3*h*_ |*J_z_*|=1/2 cannot mix with any other doublet within the ^4^I_9/2_ term, whereas in *C*_3*v*_, it can mix with both |*J_z_*|=5/2 and 7/2.

To conclude, we have prepared and fully characterized an unprecedented trigonal planar actinide triamide complex. Differences in the spectroscopic and magnetic data between **1** and [U^III^(N“)_3_] can be attributed to differences in symmetry that may be useful to consider in the future design of U^III^ SMMs with greater relaxation times. Computational analyses of **1** and [U^III^(N”)_3_] have shown only minor differences in their calculated bonding schemes, therefore, the energy gained by pyramidalization, which leads to favorable agostic M**⋅⋅⋅**Si–C_γ_ interactions in [U^III^(N“)_3_],8d, 32, 33 can be overcome by sterically demanding ligands, such as N**.

## Experimental Section

**Synthesis of 1**: THF (20 mL) was added to a precooled (−78 °C) mixture of [K{N(SiMe_2_*t*Bu)_2_}]_2_ (1.007 g, 1.5 mmol) and [U(I)_3_(THF)_4_] (0.907 g, 1 mmol). The reaction mixture was allowed to warm to RT slowly with stirring over 48 h, with precipitation of a pale solid. Volatiles were removed in vacuo, and the dark purple solid was extracted with hexanes (3×10 mL). Recrystallization from hexanes (5 mL) at −30 °C gave **1** as dark purple needles (0.605 g, 62 %).^1^H NMR (400.13 MHz, [D_6_]benzene, 25 °C, TMS): *δ*=−47.04 (br s, *ν*½=4597 Hz, 36 H; Si(C*H*_3_)_2_), 3.79 ppm (br s, *ν*½=206 Hz, 54 H; SiC(C*H*_3_)_3_); ^13^C{^1^H} NMR (100.61 MHz, [D_6_]benzene, 25 °C, TMS): *δ*=−2.13 (Si(*C*H_3_)_2_), 1.45 (Si(*C*H_3_)_2_), 18.22 (Si*C*(CH_3_)_3_), 26.40 (SiC(*C*H_3_)_3_), 31.98 ppm (Si*C*(CH_3_)_3_); ^29^Si{^1^H} NMR (79.48 MHz, [D_6_]benzene, 25 °C, TMS): *δ*=−296.04 ppm (br. s, ν1/2=73 Hz); FTIR (Nujol); 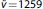 (s), 1247 (s), 1002 (s), 950 (m, asym. str., UNSi_2_), 825 (s, sym. str., UNSi_2_), 761 (s, sym. str., UNSi_2_), 655 (m), 604 (s) cm^−1^; *μ*_eff_=2.59 μ_B_ (Evans method); elemental analysis calcd for C_36_H_90_Si_6_N_3_U (971.67 g mol^−1^): C 44.5, H 9.34, N 4.33; found: C 38.29, H 9.10, N 4.22. Low carbon values were obtained upon repeating the analysis multiple times on different batches and is ascribed to **1** being a silicon-rich molecule, as was observed previously.^42^
